# Posterior Disconnection Syndrome in Early‐Stage Adult‐Onset Cerebral Adrenoleukodystrophy

**DOI:** 10.1002/acn3.70501

**Published:** 2026-08-16

**Authors:** Kazuto Katsuse, Takashi Matsukawa, Yumiko Nakamura, Kazuo Kakinuma, Shoko Ota, Ryo Hara, Saki Nakashima, Kaoru Ito, Yutaro Nakamori, Toshiyuki Kakumoto, Masashi Hamada, Shigenori Kanno, Kyoko Suzuki, Tatsushi Toda

**Affiliations:** ^1^ Department of Neurology, Graduate School of Medicine The University of Tokyo Tokyo Japan; ^2^ Department of Behavioral Neurology and Cognitive Neuroscience Tohoku University Graduate School of Medicine Sendai Japan; ^3^ Dementia Center The University of Tokyo Hospital Tokyo Japan; ^4^ Center for Higher Brain Dysfunction Support Tohoku Medical and Pharmaceutical University Hospital Sendai Japan; ^5^ National Center Hospital National Center of Neurology and Psychiatry Tokyo Japan

**Keywords:** adrenoleukodystrophy, disconnection, visual dysfunction

## Abstract

Adult‐onset cerebral adrenoleukodystrophy is potentially treatable but often difficult to recognize before advanced cerebral involvement. Herein, we describe three men with early‐stage disease who initially presented with subtle visual complaints rather than subcortical dementia. Targeted neuropsychological testing revealed higher‐order visual dysfunction and posterior interhemispheric disconnection signs, including visual‐field‐dependent reading or naming deficits and dichotic listening abnormalities. Diffusion tensor imaging showed preferential involvement of posterior callosal and visual stream‐related white‐matter pathways. These findings define a posterior disconnection phenotype of adult‐onset cerebral adrenoleukodystrophy and provide a clinically useful framework for early recognition and timely therapeutic consideration of this treatable leukodystrophy.

## 1 Introduction

Adrenoleukodystrophy (ALD) is a rare X‐linked neurological disorder caused by mutations in *ABCD1*, which encodes a peroxisomal membrane transporter whose dysfunction leads to accumulation of very‐long‐chain fatty acids (VLCFAs) [1]. ALD shows marked phenotypic heterogeneity involving the cerebrum, cerebellum, brainstem, spinal cord, and adrenal cortex, with onset from childhood to adulthood [2, 3]. Adult‐onset cerebral adrenoleukodystrophy (ACALD), accounting for approximately 21% of ALD cases in Japan, typically presents with cognitive and behavioral disturbances and often mimics psychiatric disorders or other dementias [4]. If untreated, ACALD follows a rapidly progressive inflammatory demyelinating course. Allogeneic hematopoietic stem cell transplantation is currently the only disease‐modifying treatment for ACALD, and early treatment, particularly at a Loes score ≤ 13, has been associated with stabilization of cognitive decline and improved survival, underscoring the need for timely diagnosis and intervention [5, 6].

Despite this therapeutic window, ACALD's initial clinical and neuropsychological manifestations are poorly understood. Previous reports primarily describe nonspecific subcortical dementia profiles with psychomotor slowing and deficits in attention, memory, and executive function, possibly reflecting advanced white matter involvement [7, 8, 9, 10, 11]. In contrast, ACALD's typical posterior lesion distribution, involving the parieto‐occipital white matter and splenium of the corpus callosum [5], is expected to disrupt higher‐order visual processing and posterior interhemispheric transfer. Although reduced nonverbal intelligence and memory have been reported, higher‐order visual processing and interhemispheric disconnection remain understudied [7, 8, 11]. Visual symptoms are generally reported only when they become overt, manifesting as Bálint syndrome, visual agnosia of real objects, or cortical blindness, potentially resulting in missed opportunities for timely therapeutic intervention [12]. Thus, subtle visual symptoms at onset, their neuropsychological profile, and their relationship to white matter tract involvement remain insufficiently understood.

Using targeted neuropsychological assessment and diffusion tensor imaging (DTI), we show that visual complaints, higher‐order visual dysfunction, and splenial disconnection signs can offer clinically useful clues to ACALD at a mild, potentially treatable stage, before a typical subcortical dementia profile emerges.

## 2 Methods

### 2.1 Participants

This study was approved by the Institutional Review Board of the University of Tokyo (G10073). All participants provided written informed consent. Three right‐handed patients were admitted for diagnostic evaluation of suspected ACALD. The following assessments were performed during a 3‐week hospitalization. All three patients initially presented with visual symptoms.

Case 1: A 44‐year‐old man with a doctoral‐level education developed progressive difficulty perceiving images, facial features, and fine details over 6 months. Case 2: A 26‐year‐old worker developed progressive reading difficulty over 6 months, particularly with kanji characters, and traced them with his finger to aid recognition. He also reported difficulty recognizing familiar faces and judging object texture. Case 3: A 52‐year‐old man developed visuospatial disorientation 8 months before evaluation, struggling to find his car, navigate familiar roads after landmarks had changed, and notice objects directly in front of him. Subsequently, memory impairment led to occupational difficulties and retirement. All patients repeatedly underwent ophthalmological evaluation; however, no explanatory ocular abnormality was identified; in Case 2, the symptoms were considered psychogenic.

**FIGURE 1:**
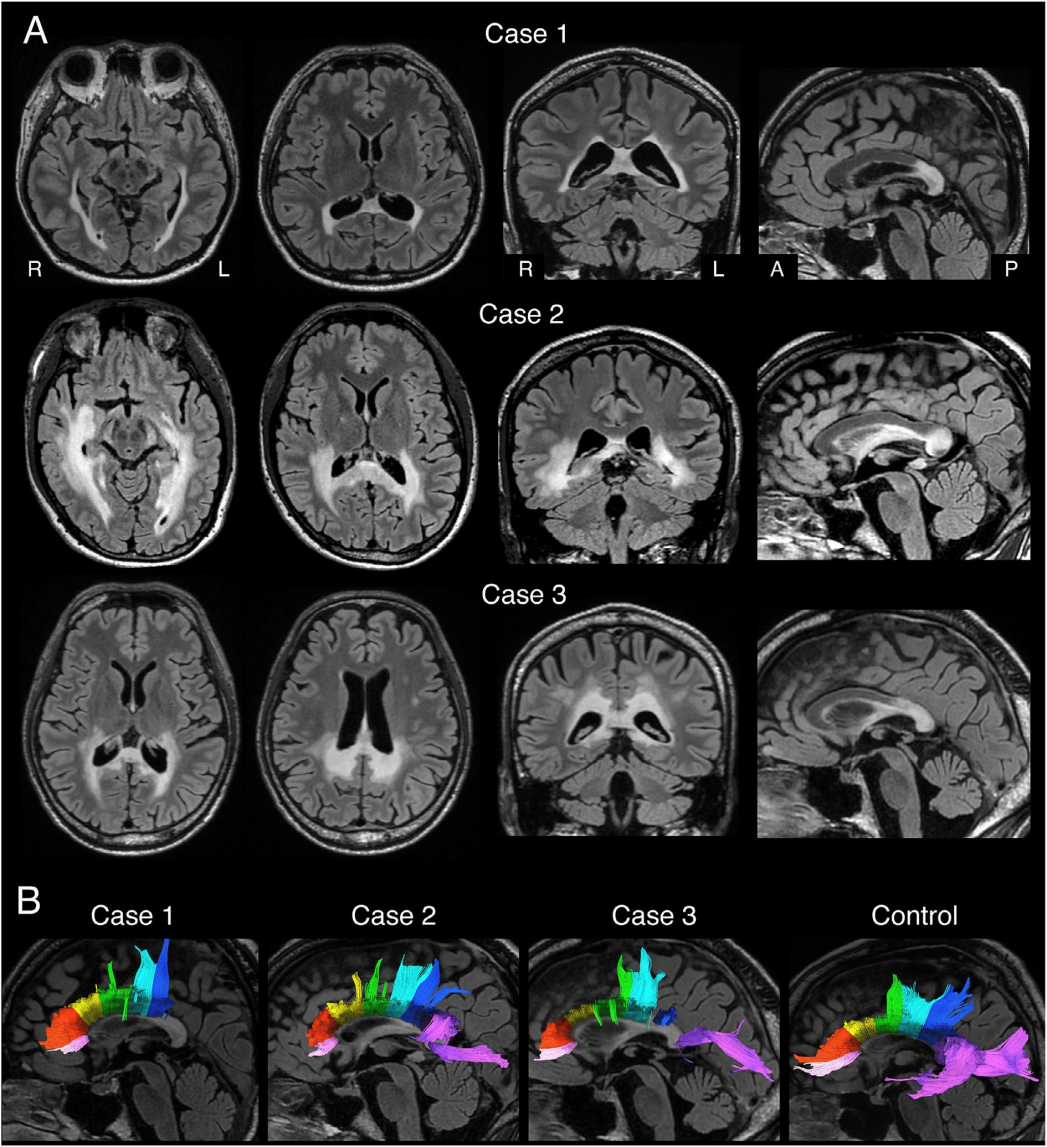
Neuroimaging findings. (A) Brain magnetic resonance imaging (MRI) showed fluid‐attenuated inversion recovery (FLAIR) hyperintense lesions in the bilateral occipitotemporal white matter and splenium of the corpus callosum in Cases 1 and 2, and in the bilateral parieto‐occipital white matter, splenium of the corpus callosum, and retrosplenial white matter and fornix in Case 3. Loes scores were 7, 12.5 and 12 for Cases 1, 2, and 3, respectively. (B) Sagittal views of three‐dimensional reconstructions of callosal fibers overlaid on midsagittal images in Cases 1–3 and a control subject with adrenomyeloneuropathy. Fibers were classified according to Witelson's scheme and color‐coded as follows: Pink, rostrum; red, genu; yellow, rostral body; green, anterior midbody; light blue, posterior midbody; dark blue, isthmus; and purple, splenium. Compared with the control subject, all three cases showed reduced splenial fibers, which was most severe in Case 1, in whom splenial fibers were not reconstructed. Case 3 also showed reduced isthmus fibers.

Near visual acuity was mildly reduced to 6/18 and 6/24 in Cases 1 and 2 but preserved at 6/9 or better in Case 3. Case 2 also showed right homonymous hemianopia. Magnetic resonance imaging (MRI) showed fluid‐attenuated inversion recovery hyperintense lesions in the posterior cerebral white matter and splenium of the corpus callosum in all patients, predominantly in the bilateral occipitotemporal white matter in Cases 1 and 2 and extending to the parieto‐occipital and retrosplenial white matter, fornix, and auditory radiations in Case 3 (Figure 1A). Plasma sphingomyelin species containing VLCFAs were markedly elevated in all cases. No patient showed clinical features of adrenal insufficiency. Sanger sequencing of *ABCD1* identified hemizygous pathogenic variants: c.832G>T, p.Glu278* in Case 1, c.760A>C, p.Thr254Pro in Case 2, and c.1628C>T, p.Pro543Leu in Case 3.

### 2.2 Neuropsychological Assessments

Targeted neuropsychological assessments were performed because all three patients initially presented with visual symptoms and posterior‐predominant lesions. General cognition, attention, executive function, language, and memory were assessed using standard Japanese neuropsychological batteries, including the Mini‐Mental State Examination, Montreal Cognitive Assessment, digit and tapping spans, Frontal Assessment Battery, Trail Making Test (TMT), and selected subsets of the Western Aphasia Battery and Wechsler Memory Scale–Revised.

Visual processing was assessed using tasks covering dorsal and ventral stream functions, based on established frameworks for posterior cortical atrophy [13]. Dorsal stream function was evaluated using dot counting and cube analysis from the Visual Object and Space Perception Battery and Behavioral Inattention Test–conventional subtest. Ventral stream function was assessed using tasks for object, color, face, and visual word processing, including line‐drawing naming from the Test of Lexical Processing in Aphasia, overlapping figure recognition [14], hue discrimination and face perception tasks from the Cortical Vision Screening Test and 10‐item kana and kanji word reading tasks. Callosal disconnection was assessed using extinction testing, tachistoscopic visual half‐field presentation, dichotic listening, tactile naming, graphesthesia, writing, and ideomotor praxis assessment (Methods S1).

### 2.3 DTI Analysis

DTI data were compared with data from 20 male controls with adrenomyeloneuropathy or Addison's disease without cerebral involvement who underwent MRI at our institution between June 2023 and November 2024. The control group's mean age was 38.0 ± 12.0 years. All participants underwent diffusion MRI using the same 3.0‐T scanner and protocol. After preprocessing with MRtrix3 and FSL, fractional anisotropy (FA) was quantified in 20 major white matter tracts from the Johns Hopkins University white matter tractography atlas (Methods S2). Tracts with *Z* scores < −2 relative to controls were considered abnormal. To further characterize posterior interhemispheric involvement, deterministic tractography of callosal fibers was performed in three cases and one representative control (a 56‐year‐old male) using DSI Studio.

## 3 Results

None of the three patients showed a typical subcortical dementia profile (Table 1). In Cases 1 and 2, cognition was largely preserved. Although TMT completion times were prolonged, the B/A ratios did not increase disproportionately, suggesting that slowed performance was influenced by visual processing rather than executive dysfunction. Although Case 3 showed severe disorientation and episodic memory impairment, verbal span and vigilance were relatively preserved, indicating selective impairment of recent memory rather than immediate memory.

**TABLE 1:** Cognitive assessment results of Cases 1–3.

		Case 1	Case 2	Case 3	Cutoff (normative mean ± SD)
**General**					
Digit span	Forward, backward	7, 5	8, 6	6, 4	
Tapping span	Forward, backward	5, 4	4, 4	3, 2	
MMSE	Total (/30)	30	28	18	
MoCA‐J	Total (/30)	29	23	10	
	Visuospatial and executive (/5)	5	2	0	
	Span/Vigilance (/3)	3	3	3	
	Memory index score (/15)	14	12	0	
FAB	Total (/18)	16	16	13	
TMT‐J	Part A, Part B (sec)	88*, 118*	378*, 528*	not feasible	45, 73
**Language**					
WAB	Spontaneous speech (/20)	20	19	20	(19.7 ± 0.6)
	Object naming (/60)	60	60	60	(59.2 ± 2.4)
	Reading (/10)	10	4.1*	10	(9.5 ± 0.8)
	Writing (/10)	10	10	10	(9.6 ± 1.0)
**Memory**					
WMS‐R	Verbal memory index	106	67*	53*	(100 ± 15.0)
	Visual memory index	97	< 50*	< 50*	(100 ± 15.0)
**Early visual processing**					
CORVIST	Near visual acuity (Rt, Lt)	6/18, 6/18	6/24, 6/24	6/9, 6/9	
	Size discrimination (/2)	2	0*	2	(1.9 ± 0.32)^a^
VOSP	Shape detection (/20)	20	19	15	15
**Dorsal stream functions**					
VOSP	Dot counting (/10)	10	9	7*	8
	Cube analysis (/10)	9	9	5*	6
BIT‐C	Line crossing (/36)	36	35	32*	34
	Star cancelation (/54)	53	47*	47*	51
	Figure and shape copying (/4)	4	4	1*	3
	Line bisection (/9)	9	8	4*	7
	Representational drawing (/3)	3	3	0*	2
**Ventral stream functions**					
TLPA	Line drawing naming (/200)	189	103*	195	(193.4 ± 5.43)
CORVIST	Unusual views (/7)^a^	1*	0*	5	(6.0 ± 0.82)^a^
	Usual views (/7)^a^	5*	5*	7	(6.9 ± 0.32)^a^
	Hue discrimination (/4)	2*	2*	4	(3.8 ± 0.63)^a^
	Face perception test 1 & 2 (/16)	6*	12*	15	(15.1 ± 1.50)^a^
Overlapping figures, total (/40)		16*	10*	30	(32.9 ± 4.40)^b^
**Reading**					
SALA	4‐mora Kanji words (/10)	10	4*	10	9
	4‐mora Kana words (/10)	10	10	10	9
	4‐mora Kana pseudowords (/14)	14	3*	NE	12
**Callosal syndromes**		Visual field (Rt, Lt)			
Extinction	Visual	−, +	Hemianopia	−, +	
Tachistoscope	Kana reading (/24)	23, 0	NE	23, 1	
	Kanji reading (/24)	22, 0	NE	24, 3	
	Line drawing naming (/20)	19, 0	NE	20, 7	
	Line drawing choice (/20)	20, 14	NE	20, 20	
		Ear (Rt, Lt)			
Extinction	Auditory	−, −	−, −	−, +	
Dichotic listening, 3‐mora words (/20)		20, 1	20, 11	NE	
		Hand (Rt, Lt)			
Extinction	Tactile	−, −	−, −	−, −	
Tactile naming	Objects (/10)	10, 10	10, 10	9, 7	
Graphesthesia	Numbers (/10)	10, 10	10, 10	8, 1	
Writing	4‐mora Kanji words (/10)	10, 9	9, 9	7, 0	
	4‐mora Kana words (/10)	10, 10	10, 10	10, 8	
Apraxia	Upper limb & tool use (/30)	30, 30	30, 30	30, 30	

*Note:* A plus sign (+) indicates the presence of extinction, and a minus sign (−) indicates its absence. Abbreviations: BIT‐C, behavioral inattention test conventional subtest; CORVIST, cortical vision screening test; FAB, frontal assessment battery; Lt, left; MMSE, mini‐mental state examination; MoCA‐J, Japanese version of the Montreal cognitive assessment; NE, not examined; Rt, right; SALA, Sophia analysis of language in aphasia (Japanese); SD, standard deviation; TLPA, test of lexical processing in aphasia (Japanese); TMT‐J, Japanese version of the trail making test; WAB, Japanese version of the western aphasia battery; WMS‐R, Wechsler memory scale‐revised (Japanese).

^a^ Normative data for the CORVIST were obtained from 10 healthy Japanese adults aged 50–76 years (mean 63.6 ± 8.5). Seven items were used in each of the Unusual Views and Usual Views subtests. The “cheese grater” item was excluded because it was unfamiliar to Japanese participants.

^b^ Normative data for the overlapping figure recognition task were derived from [14].

* Asterisks indicate scores below the cutoff.

All patients exhibited higher‐order visual dysfunction. In all cases, tapping span was reduced relative to digit span. Cases 1 and 2 showed impaired recognition of line drawings, colors, and faces; Case 2 also had pure alexia. Both performed poorly in overlapping figure recognition, with frequent shape‐related errors, indicating impaired recognition under complex conditions. In contrast, Case 3 showed a profile suggestive of dorsal stream dysfunction, including poor copying, cancelation, and spatial tasks. Signs of posterior callosal disconnection were observed in all patients. In Cases 1 and 3, tachistoscopic testing showed impaired left visual‐field reading and naming despite nearly perfect right visual‐field performance, whereas nonverbal matching was relatively preserved, even in the left visual field [15, 16]. Dichotic listening revealed a marked left‐ear disadvantage in Cases 1 and 2, and left‐sided auditory extinction was observed in Case 3. Tactile naming, graphesthesia, and writing were impaired in the left hand only in Case 3, suggesting additional involvement of more rostral callosal pathways.

DTI showed FA reduction in the forceps major in all three patients (Table 2). Cases 1 and 2 also showed abnormalities in ventral visual stream‐related tracts, including the inferior fronto‐occipital fasciculi and inferior longitudinal fasciculi, whereas Case 3 showed abnormalities in the superior longitudinal fasciculus, a dorsal visual stream‐related tract. Abnormalities also involved the hippocampal cingulum in Cases 2 and 3, as well as the cingulum within the cingulate gyrus in Case 3. Callosal tractography demonstrated marked reduction in splenial fibers in all three patients, with additional isthmus involvement in Case 3 (Figure 1B).

**TABLE 2:** Mean fractional anisotropy and corresponding *Z* scores of major white matter tracts in Cases 1–3 relative to controls.

	Control group (*n* = 20) (mean ± SD)	Case 1	Case 2	Case 3
Age	38.0 ± 12.0	44	26	52
**Tracts**	FA	*Z*	*Z*	*Z*
Forceps major	0.6962 ± 0.0217	−11.479*	−11.280*	−13.652*
Forceps minor	0.5401 ± 0.0251	−0.9435	−1.9574	−2.2438*
L ATR	0.4605 ± 0.0167	−2.4139*	−1.9047	−0.6032
L CGC	0.6682 ± 0.0309	−0.7331	−1.0402	−8.6911*
L CGH	0.5678 ± 0.0274	−0.0356	−2.5850*	−1.9522
L CST	0.6088 ± 0.0246	−1.1197	−0.8743	−1.3894
L IFOF	0.5312 ± 0.0264	−2.3768*	−5.5625*	0.0193
L ILF	0.4933 ± 0.0254	−0.9918	−6.4787*	−0.9952
L SLF	0.4872 ± 0.0209	−0.0644	−0.4269	−2.3204*
L SLF‐t	0.5387 ± 0.0356	0.0256	−4.5149*	−1.4574
L UF	0.5322 ± 0.0310	0.0636	−2.9507*	−0.1768
R ATR	0.4768 ± 0.0213	−0.9089	−1.6447	−1.1629
R CGC	0.5988 ± 0.0303	−1.4280	−1.7775	−6.6758*
R CGH	0.5506 ± 0.0361	−1.0507	−2.7584*	−4.6501*
R CST	0.6129 ± 0.0249	−1.9173	−2.1083*	−1.2028
R IFOF	0.5200 ± 0.0242	−2.4445*	−6.4312*	−1.0689
R ILF	0.5023 ± 0.0278	−1.4451	−8.3366*	−1.6592
R SLF	0.5145 ± 0.0245	−1.1399	−1.0383	−3.6909*
R SLF‐t	0.5756 ± 0.0317	0.4833	−3.7745*	−0.9991
R UF	0.5330 ± 0.0326	0.8394	−3.4782*	0.3958

Abbreviations: ATR, anterior thalamic radiation; CGC, cingulum (cingulate gyrus); CGH, cingulum (hippocampal portion); CST, corticospinal tract; FA, fractional anisotropy; IFOF, inferior fronto‐occipital fasciculus; ILF, inferior longitudinal fasciculus; L, left; R, right; SD, standard deviation; SLF, superior longitudinal fasciculus; SLF‐t, superior longitudinal fasciculus (temporal portion); UF, uncinate fasciculus.

* Asterisks indicate values with a *Z* score < −2.

## 4 Discussion

We aimed to characterize the early neuropsychological manifestations of ACALD. Our findings suggest that early‐stage ACALD with Loes scores ≤ 13 can present as subtle visual abnormalities before typical subcortical dementia or overt cortical visual syndromes emerge. Initially overlooked or misattributed, these symptoms corresponded to higher‐order visual dysfunction and splenial disconnection signs on targeted neuropsychological assessments, together with reduced FA in posterior visual and interhemispheric pathways.

These findings have three clinical implications. First, early ACALD should not be viewed as a subcortical dementia characterized by psychomotor slowing. In posterior‐predominant disease, poor performance in attention or executive tasks may reflect visuospatial dysfunction, not primary frontal‐subcortical impairment. Memory impairment, as in Case 3, may reflect anterograde episodic amnesia resulting from disruption of the hippocampal‐diencephalic‐cingulate memory network rather than attentional dysfunction [17]. Second, assessing higher‐order visual processing may detect early posterior cerebral involvement. Although visual dysfunction is recognized in childhood‐onset cerebral ALD [18, 19, 20], similar higher‐order visual symptoms are rarely reported in ACALD, suggesting under‐recognition in adults. The evaluation should include tasks sensitive to both dorsal and ventral stream dysfunctions, such as cancelation tasks and overlapping figure recognition. Third, signs of splenial disconnection may represent a specific early manifestation of ACALD, given the characteristic involvement of the splenium and adjacent posterior white matter. Tachistoscopic testing and dichotic listening may be useful for detecting impaired posterior interhemispheric transfer in ACALD.

The convergence of clinical history, neuropsychological findings, and DTI findings supports the relevance of early posterior visual and interhemispheric disconnection symptoms in ACALD. Recognizing this phenotype may prompt diagnosis before advanced dementia or overt cortical visual syndromes develop, facilitating timely intervention.

## Author Contributions

K.Kat., and T.M. conceptualized and designed the study. K.Kat., T.M., Y.N., R.H., S.N., K.I., Y.N., and T.K. acquired the data. K.Kat., K.Kak., S.O., M.H., S.K., and K.S. analyzed and interpreted the data. T.T. supervised the study. K.Kat. drafted the manuscript. All authors critically reviewed and revised the manuscript and approved the final version of the article.

## Funding

This work was supported by Japan Society for the Promotion of Science, 22K07533, 25K10746, 25K23923.

## Consent

The patients provided written informed consent to publish their anonymized data and clinical histories.

## Conflicts of Interest

The authors declare no conflicts of interest.

## Supporting information


**Methods S1**. Assessment of disconnection syndrome.
**Methods S2**. Diffusion tensor imaging processing and analysis.

## Data Availability

The data that support the findings of this study are available from the corresponding author, upon reasonable request.
