# Supplementary material for: Posterior Disconnection Syndrome in Early‐Stage Adult‐Onset Cerebral Adrenoleukodystrophy
Source: Ann Clin Transl Neurol. 2026 Aug 16;13(11):2388–94. doi: 10.1002/acn3.70501 (PMC13478642; doi:10.1002/acn3.70501)
Supplement: Supplementary file 1 [file ACN3-13-2388-s001.docx]

**Methods S1. Assessment of disconnection syndrome**

Disconnection syndrome was assessed using a multimodal battery designed to detect interhemispheric transfer deficits across the visual, auditory, tactile, language, and praxis domains. The battery included extinction testing, a computer-based tachistoscopic task, dichotic listening, tactile naming, graphesthesia, writing, and ideomotor apraxia assessment.

***Extinction***

Extinction was assessed using double simultaneous stimulation in the visual, auditory, and tactile modalities. Unilateral and bilateral stimuli were presented, and performance under bilateral stimulation was compared with that under unilateral conditions to determine whether contralesional stimuli were selectively missed. The patients responded verbally on each trial by indicating whether the stimulus was perceived on the left, right, or both sides.

***Tachistoscopic visual half-field task***

To examine lateralized visual recognition and possible callosal transfer impairment, a computerized tachistoscopic task was implemented using PsychoPy (v2023.1.0) in Cases 1 and 3. The task was not administered in Case 2 because of right homonymous hemianopia. The stimuli comprised 24 Japanese characters (kanji and kana), 20 line drawings for naming, and 20 four-alternative matching trials using the same line drawings. The stimuli were presented at an eccentricity of approximately 2.5° from central fixation on a 13-inch monitor (MacBook Air) at a viewing distance of approximately 57 cm, following a previously described method (Morihara et al., 2021). The character stimuli subtended approximately 1° × 1° of visual angle and the line drawings subtended approximately 2.5° × 2.5°. Each trial began with a central fixation cross, after which the stimulus was presented for 100 ms, a duration commonly used in divided visual-field paradigms to minimize saccadic eye movements and favor initial processing by the contralateral hemisphere (Vingerhoets et al., 2023). The patients were instructed to maintain fixation throughout the task. Depending on the stimulus condition, each patient was asked to read the presented character aloud, name the depicted object, or select the corresponding item from four alternatives.

***Dichotic listening***

In Cases 1 and 2, interhemispheric auditory processing was assessed using a dichotic listening task. Both patients had a four-frequency pure-tone average of ≤10 dB in both ears, with no interaural difference. Twenty pairs of three-mora words were presented simultaneously, with one word presented to each ear, via audiometric headphones at 45 dB above the individual hearing threshold. The patients were instructed to report both words heard on each trial.

***Tactile naming***

Tactile naming was assessed separately for each hand using ten familiar everyday objects. With vision occluded, the patients were asked to palpate each object and name it. Elementary somatosensory function was clinically confirmed before testing to ensure that impaired performance could not be attributed simply to primary sensory loss.

***Graphesthesia***

Graphesthesia was assessed separately for each hand. With the patients’ eyes closed, ten single-digit numbers (0–9) were traced on each palm in random order and the patients were asked to identify each number. Primary sensory deficits were clinically excluded prior to interpretation.

***Writing***

Writing ability was assessed to detect unilateral agraphia or other asymmetries suggestive of callosal disconnection. Writing performance for four-mora words (10 kanji and 10 kana items) selected from the D39 subset of the Sophia Analysis of Language in Aphasia was examined separately for each hand.

***Ideomotor apraxia***

Ideomotor apraxia was assessed using gesture production and the pantomime of tool use, each under verbal command and imitation conditions; for tool-use tasks, performance with actual objects was also evaluated. Ten items related to upper-limb movements and tool use were selected from the praxis subtest of the Western Aphasia Battery, with a maximum score of 30 points for each hand.

**Methods S2. Diffusion tensor imaging processing and analysis**

To identify the white matter tracts affected in the three patients with adult-onset cerebral ALD, diffusion tensor imaging (DTI) data were compared with those of a control group comprising 20 consecutive patients with adrenomyeloneuropathy or Addison’s disease without cerebral involvement who underwent MRI at our institution between June 2023 and November 2024. The mean age of the control group was 38.0 ± 12.0 years (range, 23–70 years), and all participants were male. The absence of cerebral symptoms was confirmed at the 1-year follow-up after MRI.

All 23 participants were scanned using a common protocol. DTI was performed using a 3.0-T MRI scanner (SIGNA Premier, GE Healthcare) with a 2D single-shot spin-echo echo-planar imaging sequence. The acquisition parameters were as follows: repetition time, 7500 ms; echo time, 55 ms; flip angle, 90°; slice thickness, 2.5 mm with no interslice gap. Diffusion gradients were applied along 30 directions with five non-diffusion-weighted images. Diffusion-weighted data were preprocessed using MRtrix3 (Tournier et al., 2019) and FMRIB Software Library (FSL), including denoising, Gibbs ringing correction, eddy current- and motion-induced distortion correction, and bias field correction. Brain extraction was performed using BET. Diffusion tensors were fitted voxel-wise using DTIFIT to generate fractional anisotropy (FA) maps. To minimize the effects of inter-individual misalignment, the FA maps were further processed using the Tract-Based Spatial Statistics pipeline (Smith et al., 2006) implemented in FSL. For each subject, the FA images were nonlinearly registered to the FMRIB58_FA standard-space template, and skeletonized FA maps were generated. The regions of interest (ROIs) corresponding to 20 major white matter tracts were defined based on the Johns Hopkins University white matter tractography atlas (JHU-ICBM-tracts-maxprob-thr25). These included the forceps major and minor, anterior thalamic radiation (ATR), corticospinal tract, cingulum in the cingulate gyrus (CGC), cingulum in the hippocampal region (CGH), inferior fronto-occipital fasciculus (IFOF), inferior longitudinal fasciculus (ILF), superior longitudinal fasciculus (SLF), superior longitudinal fasciculus (temporal portion), and uncinate fasciculus (UF), bilaterally where applicable. The mean FA values were extracted from each tract on the skeleton using a threshold of FA > 0.2. Mean FA values were standardized to *Z* scores relative to the control group (*Z* = [patient − control mean] / standard deviation). Tracts with *Z* < −2 were considered to show significant FA reduction.

To further characterize interhemispheric white matter involvement, tractography of the callosal fibers was performed using DSI Studio (Morihara et al., 2021). The analysis was performed in Cases 1–3, and in one representative subject from the control group (a 56-year-old man with adrenomyeloneuropathy). Diffusion data were reconstructed using generalized q-sampling imaging with a diffusion sampling length ratio of 1.25 (Yeh et al., 2010). Fibers traversing a manually defined corpus callosum region of interest on the midsagittal plane were reconstructed. Deterministic fiber tracking was then performed using an anisotropy threshold of 0.20–0.40, an angular threshold of 50°, and a step size of 1 mm. Streamlines shorter than 30 mm or longer than 200 mm were discarded, and a total of 10,000 tracts were generated.

**References**

Morihara K, Kakinuma K, Kobayashi E, et al. Improvement in callosal disconnection syndrome with recovery of callosal connectivity. *Neurocase*. 2021;27(3):323-331. doi:10.1080/13554794.2021.1959935

Smith SM, Jenkinson M, Johansen-Berg H, et al. Tract-based spatial statistics: voxelwise analysis of multi-subject diffusion data. *Neuroimage*. 2006;31(4):1487-1505. doi:10.1016/j.neuroimage.2006.02.024

Tournier JD, Smith R, Raffelt D, et al. MRtrix3: A fast, flexible and open software framework for medical image processing and visualisation. *Neuroimage*. 2019;202:116137. doi:10.1016/j.neuroimage.2019.116137

Vingerhoets G, Verhelst H, Gerrits R, et al. Laterality indices consensus initiative (LICI): A Delphi expert survey report on recommendations to record, assess, and report asymmetry in human behavioural and brain research. *Laterality*. 2023;28(2-3):122-191. doi:10.1080/1357650X.2023.2199963
